# Crystal structure of bis­(di­methyl­ammonium) hexa­aqua­nickel(II) bis­(sulfate) dihydrate

**DOI:** 10.1107/S160053681402234X

**Published:** 2014-10-24

**Authors:** Peter Held

**Affiliations:** a Institut für Kristallographie, Universität zu Köln, Greinstrasse 6, D-50939 Köln, Germany

**Keywords:** crystal structure, di­methyl­ammonium salt, hexa­aqua­nickel(II) salt, sulfate, hydrogen bonding

## Abstract

In the title salt, (C_2_H_8_N)_2_[Ni(H_2_O)_6_)](SO_4_)_2_·2H_2_O, the Ni^II^ cation is located on a centre of inversion and exhibits a slightly distorted octa­hedral arrangement of water mol­ecules. The Ni—O bond lengths in the complex [Ni(H_2_O)_6_]^2+^ cation show a distribution as in the related Tutton salt (NH_4_)_2_[Ni(H_2_O)_6_](SO_4_)_2_, but are longer in average [2.056 (13) *versus* 2.037 (12) Å]. The noncoordinating water mol­ecules and di­methyl­ammonium cations connect the sulfate and [Ni(H_2_O)_6_]^2+^ octa­hedra *via* O—H⋯O and N—H⋯O hydrogen bonds from weak up to medium strength into a three-dimensional framework whereby the complex metal cations and sulfate anions are arranged in sheets parallel (001).

## Abstract 2

In the salt bis­(di­methyl­ammonium) hexa­aqua­nickelate(II) bis­(sulfate) dihydrate, the Ni^II^ cation is located on a centre of inversion and exhibits a slightly distorted octa­hedral arrangement of water mol­ecules. The noncoordinating water mol­ecules and di­methyl­ammonium cations connect the sulfate and [Ni(H_2_O)_6_]^2+^ octa­hedra *via* O—H⋯O and N—H⋯O hydrogen bonds into a three-dimensional framework.

## Chemical context   

In the course of a systematic search for new ‘double salts’ of simple secondary amines and divalent cations of various inorganic acids, the structure of [(CH_3_)_2_NH_2_][Cu(HSO_4_)(SO_4_)(H_2_O)_4_] has been described previously (Held, 2014 ▶). In continuation of these studies, copper(II) was replaced by nickel(II), yielding crystals of the title compound with composition (C_2_H_8_N)_2_[Ni(H_2_O)_6_)](SO_4_)_2_·2H_2_O.
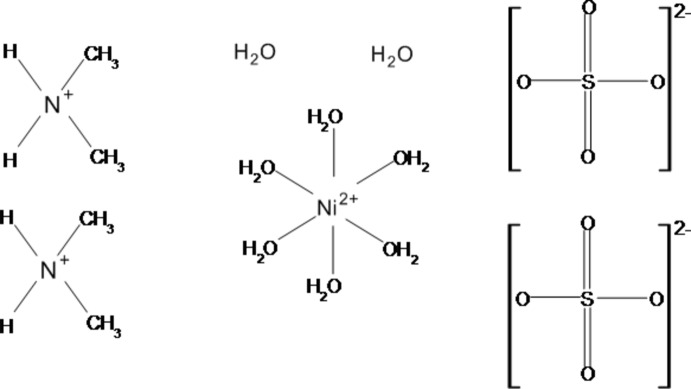


## Structural commentary   

**Figure 1:**
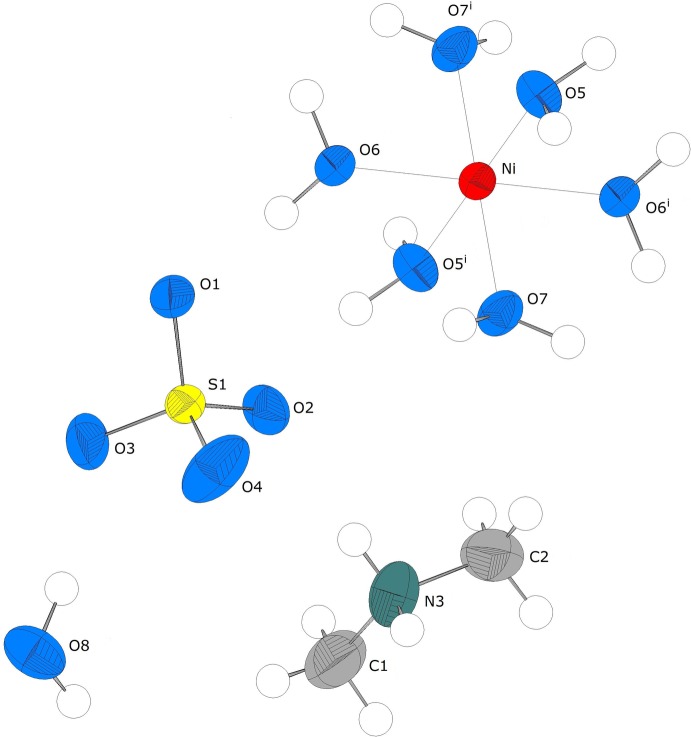
The mol­ecular entities in the structure of the title compound, showing the atom-numbering scheme. Displacement ellipsoids are drawn at the 50% probability level. [Symmetry code: (i) −*x*, −*y* + 1, −*z* − 1.]

The asymmetric unit of the title compound consists of one [NH_2_(CH_3_)]^+^ cation, one Ni^2+^ cation situated on an inversion centre (Wyckoff position 4*a*), one SO_4_^2−^ anion and four water mol­ecules, one of which is not coordinating to the metal cation (Fig. 1 ▶). The Ni^II^ cation exhibits a slightly distorted octa­hedral arrangement of the water mol­ecules. The Ni—O distances show the same bond lengths distribution [mean 2.055 (12) Å], as in the related Tutton salt (NH_4_)_2_[Ni(H_2_O)_6_](SO_4_)_2_ (Grimes *et al.*, 1963 ▶), but are slightly longer (Δ*d* = 0.02 Å). The Ni^II^ cation reaches an overall bond valence sum (Brown & Altermatt, 1985 ▶) of 2.03 valence units. The S—O distances are nearly equal [mean 1.463 (8) Å], however, the O—S—O angles vary clearly [average bond angle 109.5 (8)°].

## Supra­molecular features   

**Figure 2:**
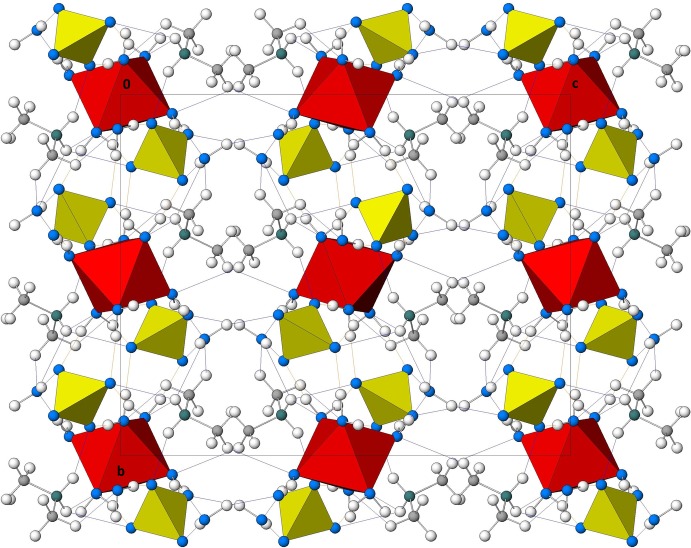
(100)-projection of the crystal structure of the title compound. Colour scheme: (SO_4_) tetra­hedra yellow, [Ni(OH_2_)_6_] octa­hedra red, O blue, N green, C grey and H colourless. H⋯O bonds up to 1.8 Å are given as orange dashed lines and from 1.85 to 2.7 Å as light-blue dashed lines.

Hydrogen bonds of weak up to medium strength involving coordinating and noncoordinating water mol­ecules as donor groups and O atoms of the sulfate anions as acceptor groups inter­connect neighbouring [Ni(H_2_O)_6_]^2+^ octa­hedra. Together with relatively weaker N—H⋯O hydrogen bonds of the ammonium H atoms to sulfate anions, a three-dimensional framework is formed with pronounced formation of sheets of complex metal cations and sulfate anions parallel (001) (Table 1 ▶ and Fig. 2 ▶).

## Synthesis and crystallization   

The title compound was obtained by reaction of an aqueous solution of nickel(II) sulfate with di­methyl­amine and sulfuric acid (18 mol l^−1^) in a stoichiometric ratio of 1:2:1. The resulting solution was kept at room temperature by cooling. The title compound crystallized by slow evaporation of the solvent at room temperature in form of light-green crystals with dimensions up to 4 mm within 12 weeks.

## Refinement   

Details of structure refinement are given in Table 2 ▶. All H atoms were clearly discernible from difference Fourier maps. However, riding-model contraints were applied to all H atoms in the least-squares refinement, with C—H = 0.96 Å and *U*_iso_(H) = 1.5*U*_eq_(C) for methyl H atoms, and N—H = 0.90 Å and *U*_iso_(H) = 1.2*U*_eq_(N) for ammonium H atoms. The H atoms of water mol­ecules were refined with a distance restraint of O—H = 0.98 Å and individual *U*_iso_ values for each H atom.

## Supplementary Material

Crystal structure: contains datablock(s) I, global. DOI: 10.1107/S160053681402234X/wm5074sup1.cif

Structure factors: contains datablock(s) I. DOI: 10.1107/S160053681402234X/wm5074Isup2.hkl

CCDC reference: 1028557

Additional supporting information:  crystallographic information; 3D view; checkCIF report

## Figures and Tables

**Table 1:** Hydrogen-bond geometry (, )

*D*H*A*	*D*H	H*A*	*D* *A*	*D*H*A*
O5H51O2^i^	0.97(1)	1.77(2)	2.727(5)	166(5)
O5H52O8	0.98(1)	1.84(1)	2.814(6)	176(7)
O6H61O3^ii^	0.97(1)	1.73(2)	2.689(5)	169(6)
O6H62O1	0.98(1)	1.78(2)	2.731(5)	164(5)
O7H71O4^iii^	0.97(1)	1.78(2)	2.730(6)	164(5)
O7H72O1^iv^	0.98(1)	1.78(2)	2.745(5)	173(7)
O8H81O3^iii^	0.98(1)	2.01(2)	2.962(6)	166(6)
O8H82O2^v^	0.98(1)	1.93(3)	2.856(6)	158(7)
N3H3*B*O4^vi^	0.90	2.02	2.835(7)	151
N3H3*A*O6^iv^	0.90	2.65	3.274(6)	127

Symmetry codes: (i) 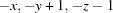; (ii) 
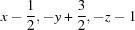
; (iii) 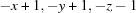; (iv) 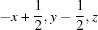; (v) 
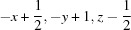
; (vi) 
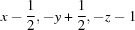
.

**Table 2:** Experimental details

Crystal data	
Chemical formula	(C_2_H_8_N)_2_[Ni(H_2_O)_6_](SO_4_)_2_2H_2_O
*M*_r_	487.13
Crystal system, space group	Orthorhombic, *P* *b* *c* *a*
Temperature (K)	295
*a*, *b*, *c* ()	8.9363(6), 13.2370(8), 16.4810(14)
*V* (^3^)	1949.5(2)
*Z*	4
Radiation type	Mo *K*
(mm^1^)	1.28
Crystal size (mm)	0.29 0.27 0.26

Data collection	
Diffractometer	EnrafNonius MACH3
Absorption correction	scan (North *et al.*, 1968 ▶)
*T*_min_, *T*_max_	0.935, 0.999
No. of measured, independent and observed [*I* > 2(*I*)] reflections	4902, 1719, 962
*R*_int_	0.107
(sin /)_max_ (^1^)	0.595

Refinement	
*R*[*F* ^2^ > 2(*F* ^2^)], *wR*(*F* ^2^), *S*	0.040, 0.123, 1.07
No. of reflections	1719
No. of parameters	148
No. of restraints	8
H-atom treatment	H atoms treated by a mixture of independent and constrained refinement
_max_, _min_ (e ^3^)	0.44, 0.41

Computer programs: *CAD-4 Software* (EnrafNonius, 1998 ▶), *MolEN* (Fair, 1990 ▶), *SIR97* (Altomare *et al.*, 1999 ▶), *ATOMS* (Dowty, 2011 ▶), *SHELXL97* (Sheldrick, 2008 ▶) and *publCIF* (Westrip, 2010 ▶).

## supplementary crystallographic information

### Crystal data

**Table:**

(C_2_H_8_N)_2_[Ni(H_2_O)_6_](SO_4_)_2_·2H_2_O	*F*(000) = 1032
*M**_r_* = 487.13	*D*_x_ = 1.660 Mg m^−^^3^
Orthorhombic, *P**b**c**a*	Mo *K*α radiation, λ = 0.71073 Å
Hall symbol: -P 2ac 2ab	Cell parameters from 25 reflections
*a* = 8.9363 (6) Å	θ = 5.5–10.1°
*b* = 13.2370 (8) Å	µ = 1.28 mm^−^^1^
*c* = 16.4810 (14) Å	*T* = 295 K
*V* = 1949.5 (2) Å^3^	Prism, light green
*Z* = 4	0.29 × 0.27 × 0.26 mm

### Data collection

**Table:**

Enraf–Nonius MACH3 diffractometer	962 reflections with *I* > 2σ(*I*)
Radiation source: fine-focus sealed tube	*R*_int_ = 0.107
Graphite monochromator	θ_max_ = 25.0°, θ_min_ = 2.5°
/w2/q scans	*h* = −10→10
Absorption correction: ψ scan (North *et al.*, 1968)	*k* = −15→15
*T*_min_ = 0.935, *T*_max_ = 0.999	*l* = −19→19
4902 measured reflections	3 standard reflections every 100 reflections
1719 independent reflections	intensity decay: 0.3%

### Refinement

**Table:**

Refinement on *F*^2^	Secondary atom site location: difference Fourier map
Least-squares matrix: full	Hydrogen site location: inferred from neighbouring sites
*R*[*F*^2^ > 2σ(*F*^2^)] = 0.040	H atoms treated by a mixture of independent and constrained refinement
*wR*(*F*^2^) = 0.123	*w* = 1/[σ^2^(*F*_o_^2^) + (0.0578*P*)^2^ + 0.0324*P*] where *P* = (*F*_o_^2^ + 2*F*_c_^2^)/3
*S* = 1.07	(Δ/σ)_max_ < 0.001
1719 reflections	Δρ_max_ = 0.44 e Å^−^^3^
148 parameters	Δρ_min_ = −0.41 e Å^−^^3^
8 restraints	Extinction correction: *SHELXL97* (Sheldrick, 2008), Fc^*^=kFc[1+0.001xFc^2^λ^3^/sin(2θ)]^-1/4^
Primary atom site location: structure-invariant direct methods	Extinction coefficient: 0.0267 (19)

### Special details

**Table:**

Experimental. A suitable single-crystal was carefully selected under a polarizing microscope and mounted in a glass capillary.
Geometry. All e.s.d.'s (except the e.s.d. in the dihedral angle between two l.s. planes) are estimated using the full covariance matrix. The cell e.s.d.'s are taken into account individually in the estimation of e.s.d.'s in distances, angles and torsion angles; correlations between e.s.d.'s in cell parameters are only used when they are defined by crystal symmetry. An approximate (isotropic) treatment of cell e.s.d.'s is used for estimating e.s.d.'s involving l.s. planes.
Refinement. Refinement of *F*^2^ against ALL reflections. The weighted *R*-factor *wR* and goodness of fit *S* are based on *F*^2^, conventional *R*-factors *R* are based on *F*, with *F* set to zero for negative *F*^2^. The threshold expression of *F*^2^ > σ(*F*^2^) is used only for calculating *R*-factors(gt) *etc*. and is not relevant to the choice of reflections for refinement. *R*-factors based on *F*^2^ are statistically about twice as large as those based on *F*, and *R*-factors based on ALL data will be even larger.

### Fractional atomic coordinates and isotropic or equivalent isotropic displacement parameters (Å^2^)

**Table:**

	*x*	*y*	*z*	*U*_iso_*/*U*_eq_	
Ni	0.0000	0.5000	−0.5000	0.0269 (3)	
S1	0.44455 (15)	0.65768 (10)	−0.40485 (8)	0.0314 (4)	
O1	0.3690 (4)	0.7003 (3)	−0.4766 (2)	0.0446 (11)	
O2	0.3354 (4)	0.6104 (3)	−0.3508 (2)	0.0424 (11)	
O3	0.5247 (5)	0.7376 (3)	−0.3626 (3)	0.0617 (13)	
O4	0.5509 (6)	0.5805 (3)	−0.4313 (3)	0.0764 (16)	
O5	−0.0494 (5)	0.4469 (3)	−0.6145 (2)	0.0382 (10)	
H51	−0.150 (3)	0.420 (4)	−0.619 (3)	0.06 (2)*	
H52	0.023 (7)	0.401 (5)	−0.639 (5)	0.12 (3)*	
O6	0.1383 (4)	0.6054 (3)	−0.5541 (2)	0.0339 (9)	
H61	0.090 (6)	0.656 (3)	−0.588 (3)	0.07 (2)*	
H62	0.209 (5)	0.639 (4)	−0.518 (3)	0.07 (2)*	
O7	0.1783 (4)	0.4042 (3)	−0.4930 (2)	0.0379 (9)	
H71	0.264 (4)	0.415 (5)	−0.528 (3)	0.07 (2)*	
H72	0.169 (9)	0.3310 (10)	−0.490 (4)	0.11 (3)*	
O8	0.1689 (5)	0.3224 (4)	−0.6862 (2)	0.0535 (12)	
H81	0.275 (2)	0.311 (5)	−0.676 (4)	0.10 (3)*	
H82	0.177 (10)	0.361 (5)	−0.736 (3)	0.12 (3)*	
N3	0.0336 (6)	0.1126 (4)	−0.6424 (3)	0.0537 (15)	
H3A	0.1022	0.1550	−0.6213	0.064*	
H3B	0.0189	0.0628	−0.6061	0.064*	
C1	0.0946 (11)	0.0689 (5)	−0.7152 (5)	0.097 (3)	
H1A	0.1864	0.0346	−0.7027	0.145*	
H1B	0.1138	0.1214	−0.7541	0.145*	
H1C	0.0243	0.0216	−0.7375	0.145*	
C2	−0.1071 (8)	0.1681 (6)	−0.6520 (5)	0.071 (2)	
H2A	−0.1374	0.1951	−0.6005	0.106*	
H2B	−0.1830	0.1231	−0.6720	0.106*	
H2C	−0.0930	0.2223	−0.6899	0.106*	

### Atomic displacement parameters (Å^2^)

**Table:**

	*U*^11^	*U*^22^	*U*^33^	*U*^12^	*U*^13^	*U*^23^
Ni	0.0289 (5)	0.0233 (5)	0.0284 (5)	0.0004 (5)	−0.0020 (4)	0.0003 (5)
S1	0.0293 (7)	0.0309 (7)	0.0340 (8)	−0.0008 (6)	−0.0010 (6)	0.0055 (6)
O1	0.055 (3)	0.040 (2)	0.038 (2)	−0.009 (2)	−0.0177 (19)	0.0099 (18)
O2	0.031 (2)	0.053 (3)	0.043 (2)	−0.0083 (19)	0.0059 (19)	0.007 (2)
O3	0.083 (3)	0.054 (3)	0.047 (3)	−0.033 (3)	−0.027 (2)	0.015 (2)
O4	0.066 (3)	0.059 (3)	0.104 (4)	0.026 (3)	0.040 (3)	0.029 (3)
O5	0.032 (2)	0.045 (2)	0.038 (2)	−0.005 (2)	−0.0017 (19)	−0.0058 (19)
O6	0.036 (2)	0.029 (2)	0.037 (2)	−0.0031 (18)	−0.0059 (18)	0.0067 (18)
O7	0.032 (2)	0.032 (2)	0.050 (3)	0.0070 (18)	0.008 (2)	0.007 (2)
O8	0.041 (3)	0.080 (3)	0.040 (3)	−0.003 (3)	0.000 (2)	−0.008 (3)
N3	0.069 (4)	0.041 (3)	0.051 (3)	0.004 (3)	−0.016 (3)	−0.005 (3)
C1	0.147 (9)	0.049 (5)	0.094 (7)	0.008 (5)	0.065 (6)	0.013 (4)
C2	0.051 (4)	0.078 (6)	0.082 (5)	−0.001 (4)	0.011 (4)	0.021 (5)

### Geometric parameters (Å, º)

**Table:**

Ni—O7^i^	2.040 (3)	O7—H71	0.974 (10)
Ni—O7	2.040 (3)	O7—H72	0.975 (10)
Ni—O5	2.061 (4)	O8—H81	0.975 (10)
Ni—O5^i^	2.061 (4)	O8—H82	0.976 (10)
Ni—O6	2.066 (4)	N3—C1	1.440 (8)
Ni—O6^i^	2.066 (4)	N3—C2	1.464 (8)
S1—O3	1.455 (4)	N3—H3A	0.9000
S1—O4	1.461 (4)	N3—H3B	0.9000
S1—O2	1.461 (4)	C1—H1A	0.9600
S1—O1	1.474 (4)	C1—H1B	0.9600
O5—H51	0.974 (10)	C1—H1C	0.9600
O5—H52	0.976 (10)	C2—H2A	0.9600
O6—H61	0.973 (10)	C2—H2B	0.9600
O6—H62	0.976 (10)	C2—H2C	0.9600
			
O7^i^—Ni—O7	180.0 (2)	Ni—O6—H62	116 (4)
O7^i^—Ni—O5	89.60 (16)	H61—O6—H62	109 (5)
O7—Ni—O5	90.40 (16)	Ni—O7—H71	119 (4)
O7^i^—Ni—O5^i^	90.40 (16)	Ni—O7—H72	123 (5)
O7—Ni—O5^i^	89.60 (16)	H71—O7—H72	104 (6)
O5—Ni—O5^i^	180.0	H81—O8—H82	99 (6)
O7^i^—Ni—O6	91.33 (15)	C1—N3—C2	115.8 (6)
O7—Ni—O6	88.67 (15)	C1—N3—H3A	108.3
O5—Ni—O6	87.90 (15)	C2—N3—H3A	108.3
O5^i^—Ni—O6	92.10 (15)	C1—N3—H3B	108.3
O7^i^—Ni—O6^i^	88.67 (15)	C2—N3—H3B	108.3
O7—Ni—O6^i^	91.33 (15)	H3A—N3—H3B	107.4
O5—Ni—O6^i^	92.10 (15)	N3—C1—H1A	109.5
O5^i^—Ni—O6^i^	87.90 (15)	N3—C1—H1B	109.5
O6—Ni—O6^i^	180.00 (19)	H1A—C1—H1B	109.5
O3—S1—O4	109.3 (3)	N3—C1—H1C	109.5
O3—S1—O2	110.4 (3)	H1A—C1—H1C	109.5
O4—S1—O2	108.4 (3)	H1B—C1—H1C	109.5
O3—S1—O1	109.3 (2)	N3—C2—H2A	109.5
O4—S1—O1	109.0 (3)	N3—C2—H2B	109.5
O2—S1—O1	110.3 (2)	H2A—C2—H2B	109.5
Ni—O5—H51	113 (3)	N3—C2—H2C	109.5
Ni—O5—H52	117 (5)	H2A—C2—H2C	109.5
H51—O5—H52	110 (6)	H2B—C2—H2C	109.5
Ni—O6—H61	117 (4)		

Symmetry code: (i) −*x*, −*y*+1, −*z*−1.

### Hydrogen-bond geometry (Å, º)

**Table:**

*D*—H···*A*	*D*—H	H···*A*	*D*···*A*	*D*—H···*A*
O5—H51···O2^i^	0.97 (1)	1.77 (2)	2.727 (5)	166 (5)
O5—H52···O8	0.98 (1)	1.84 (1)	2.814 (6)	176 (7)
O6—H61···O3^ii^	0.97 (1)	1.73 (2)	2.689 (5)	169 (6)
O6—H62···O1	0.98 (1)	1.78 (2)	2.731 (5)	164 (5)
O7—H71···O4^iii^	0.97 (1)	1.78 (2)	2.730 (6)	164 (5)
O7—H72···O1^iv^	0.98 (1)	1.78 (2)	2.745 (5)	173 (7)
O8—H81···O3^iii^	0.98 (1)	2.01 (2)	2.962 (6)	166 (6)
O8—H82···O2^v^	0.98 (1)	1.93 (3)	2.856 (6)	158 (7)
N3—H3*B*···O4^vi^	0.90	2.02	2.835 (7)	151
N3—H3*A*···O6^iv^	0.90	2.65	3.274 (6)	127

Symmetry codes: (i) −*x*, −*y*+1, −*z*−1; (ii) *x*−1/2, −*y*+3/2, −*z*−1; (iii) −*x*+1, −*y*+1, −*z*−1; (iv) −*x*+1/2, *y*−1/2, *z*; (v) −*x*+1/2, −*y*+1, *z*−1/2; (vi) *x*−1/2, −*y*+1/2, −*z*−1.
